# Oxidative stress biomarkers in treatment-responsive and treatment-resistant schizophrenia patients

**DOI:** 10.47626/2237-6089-2020-0078

**Published:** 2021-12-10

**Authors:** Patrick Buosi, Fábio Aparecido Borghi, Angélica Marta Lopes, Isabela da Silva Facincani, Rafael Fernandes-Ferreira, Camila Ive Ferreira Oliveira-Brancati, Tayanne Silva do Carmo, Dorotéia Rossi Silva Souza, Danilo Grünig Humberto da Silva, Eduardo Alves de Almeida, Gerardo Maria de Araújo

**Affiliations:** 1 Hospital de Base Faculdade de Medicina de São José do Rio Preto São José do Rio Preto SP Brazil Hospital de Base, Faculdade de Medicina de São José do Rio Preto (FAMERP), São José do Rio Preto, SP, Brazil.; 2 FAMERP São José do Rio Preto SP Brazil FAMERP, São José do Rio Preto, SP, Brazil.; 3 Universidade Estadual de Campinas Campinas SP Brazil Universidade Estadual de Campinas (UNICAMP), Campinas, SP, Brazil.; 4 Universidade Paulista São José do Rio Preto SP Brazil Universidade Paulista (UNIP), São José do Rio Preto, SP, Brazil.; 5 Instituto de Biociencias Letras e Ciencias Exatas Universidade Estadual Paulista São José do Rio Preto SP Brazil Instituto de Biociencias Letras e Ciencias Exatas Campus de Sao Jose do Rio Preto (IBILCE), Universidade Estadual Paulista, São José do Rio Preto Campus (UNESP), São José do Rio Preto, SP, Brazil.; 6 Departamento de Ciências Naturais Universidade Regional de Blumenau Blumenau SC Brazil Departamento de Ciências Naturais, Universidade Regional de Blumenau (FURB), Blumenau, SC, Brazil.

**Keywords:** Psychotic disorders, oxidative stress, antioxidants, free radicals, treatment resistance

## Abstract

**Introduction:**

Schizophrenia is a complex psychiatric disorder that affects approximately twenty million people worldwide. Various factors have been associated with the physiopathology of this disease such as oxidative stress, which is an imbalance between pro-oxidant and antioxidant molecules.

**Objective:**

This study evaluated the association between biomarkers of oxidative stress and response to pharmacological treatment among patients with schizophrenia in the context of their clinical information, demographic data, and lifestyle.

**Methods:**

A total of 89 subjects were included, 26 of whom were treatment-responsive schizophrenia patients (Group 1), 27 treatment-resistant schizophrenia patients (Group 2), and 36 healthy controls (Group 3). All of the subjects completed a questionnaire to provide clinical and demographic data, and all provided peripheral blood samples. The oxidative stress markers analyzed using spectrophotometry were catalase (CAT), superoxide dismutase (SOD), glutathione peroxidase (GPx), total glutathione (GSH-t), malondialdehyde (MDA), and Trolox-equivalent antioxidant capacity (TEAC; p < 0.05).

**Results:**

When all schizophrenia patients (G1 + G2) were compared to the control group, SOD levels were found to be lower among schizophrenia patients (p < 0.0001), while MDA and CAT levels were higher (p < 0.0001 and p = 0.0191, respectively). GPx, GSH-t, and TEAC levels were similar in all three groups (p > 0.05).

**Conclusion:**

Lower SOD levels and higher MDA and CAT levels indicate oxidative damage in schizophrenia patients, regardless of their response to pharmacological treatment. Smoking is associated with oxidative stress, in addition, a family history of the disease was also found to be correlated with cases of schizophrenia, which reflects the relevance of genetics in disease development.

## Introduction

Schizophrenia is a complex, chronic, and progressive psychiatric disorder that affects approximately twenty million people worldwide, thus generating important economic impacts on society. Its onset typically occurs in late adolescence or early adulthood, a period which represents the start of working age.^1 - 3^ Patients may exhibit symptoms classified as positive (delirium, hallucinations, and disorganized behavior) or negative (a lack of movement, anhedonia, and flat affect); these symptoms can be evaluated using the Positive and Negative Syndrome Scale (PANSS).^4 - 8^ Schizophrenia treatment requires a broad-scale approach with a multidisciplinary team; psychotic symptoms are treated using antipsychotics, but, despite their efficacy, one third of schizophrenia patients fail to respond to pharmacological treatment and are therefore classified as treatment-resistant.^9 - 11^

Various factors may influence manifestation of symptoms and patient response to pharmacological treatment, including environmental, genetic, and biochemical conditions.^12^ Oxidative stress, which is understood as an imbalance between pro-oxidant and antioxidant molecules, is associated with schizophrenia progression, as well as with the development of other neurodegenerative diseases such as Alzheimer’s and Parkinson’s diseases.^11 , 13 - 15^ In this case, progression is stimulated by an increase in reactive oxygen species (ROS) production, decreased antioxidant defense, or a combination of the two.^13^

The increase in ROS production may occur in the brain as a consequence of high oxygen saturation resulting from intense neuronal metabolic activity that damages the tissues, lipid membranes, proteins, enzymes, and DNA.^16 - 19^ In this case, lipid peroxidation may generate a bioproduct such as malondialdehyde (MDA), which can be used as an oxidative biomarker.^16 - 18 , 20 , 21^ In addition, Trolox-equivalent antioxidant capacity (TEAC) evaluates total (enzymatic and non-enzymatic) antioxidant efficiency.^17 , 21 , 22^ Antioxidants inhibit the damaging reactions caused by ROS and may be represented by catalase (CAT), superoxide dismutase (SOD), glutathione peroxidase (GPx), or total glutathione (GSH-t), the last of which consists of glutathione in its reduced (GSH) and oxidized (GSSG) states. These antioxidants protect the body from free radicals and maintain redox balance inside cells.^14 , 23 , 24^

In this context, oxidative stress may be involved in patient physiopathology and response to pharmacological treatment in cases of schizophrenia, although additional information on these associations is needed, as is clarification regarding the relationships between oxidative stress and pharmacological treatment response, PANSS symptomology, clinical profile, sociodemographics, and lifestyle.

## Methods

A total of 89 adult subjects of both genders were included and divided into three groups: Group 1 (G1) comprised 26 treatment-responsive schizophrenia patients; Group 2 (G2) comprised treatment-resistant schizophrenia patients; and Group 3 (G3) comprised healthy controls.

The patients included in the study had been receiving treatment at a psychiatric disorder ambulatory care center at the São José do Rio Preto Medical School Teaching Hospital (HB-FAMPERB) for at least twelve months and were selected through convenience sampling by the local psychiatric team between October 2014 and March 2015. Patients received their schizophrenia diagnosis based on the DSM-IV criteria.^25^ The study coordinator was a psychiatrist specialized in neuroscience. All of the psychiatrists involved in the study work in clinical practice and/or had participated in prior studies on schizophrenia.

The treatment-resistant schizophrenia patients (G2) were classified in accordance with the criteria proposed by Kane et al.^26^ and the International Psychopharmacology Algorithm Project (IPAP) algorithms.^27^ According to these criteria, patients were defined as treatment-resistant when they exhibited persistent and moderate to severe positive psychotic symptoms, even when on the recommended doses of antipsychotics (doses equivalent to ≥ 400mg/day of chlorpromazine) for an appropriate amount of time (four to six weeks) and after having tried treatment episodes with at least two adequate antipsychotic drugs. In this case, the patient is considered eligible for treatment with clozapine at doses ranging from 300 mg/day to 900 mg/day. In addition, the patients were evaluated using a version of the PANSS that had been translated into Portuguese, adapted, and verified. It consisted of 30 items separated into positive, negative, and general psychopathology subscales in order to detect patients’ predominant symptomology.^28^

The control group (G3) was recruited from voluntary blood donors at the study center’s blood bank. These individuals were assessed by the principal psychiatrist, a professor from the local institution, using the appropriate structured clinical interview (SCID-DSM-IV) during their blood donation session in order to reduce sample bias. Individuals were included when the interview ruled out the possibility of any psychiatric disorders. This group was selected to match the patient groups in terms of gender and age.

The exclusion criteria consisted of use of nutritional supplements, opioids, anti-inflammatories, hydroxycarbamide, aspirin, antibiotics, or vitamins within the preceding 24 hours, pregnancy, a blood transfusion within the 120 days prior to the blood sample collection, other serious clinical or neurological diseases, and psychotic disorders other than schizophrenia. Patients who failed to provide consent or to comply with treatment were also excluded.

All of the study subjects completed a clinical and demographic questionnaire and provided a peripheral blood sample. For the oxidative stress biomarker analysis, the blood samples were collected in tubes containing EDTA anticoagulant in order to obtain the cell lysate. After centrifugation at 4°C, the plasma was discarded, and the remaining content (red cell concentrate) was washed three times in a 0.09% saline solution to obtain the hemolysate with a 20:1 dilution ratio in ultrapure water. The material was aliquoted and frozen at -80°C until analysis.

The SOD activity analysis consisted of reduction of cytochrome c by the superoxide radical (O_2_^●ˉ^), generated by the xanthine/xanthine oxidase enzyme system, which results in an increase in absorbance at 500 nm.^29^ The cytochrome c reduction rate is inhibited as a result of the addition of SOD, which competes with cytochrome c for O_2_^●ˉ^, generating hydrogen peroxide (H_2_O_2_). CAT activity was determined based on the rate of H_2_O_2_ decomposition produced by CAT, with a decrease in absorbance at 240 nm.^30^ GPx was analyzed based on absorbance at 340 nm, resulting from reduced consumption of nicotinamide adenine dinucleotide phosphate (NADPH) by glutathione reductase (GR) during GSSG reduction to GSH.^31^ 5-mercapto-2-nitrobenzoic acid (Nbs), a derivative produced by the oxidation of GSH, was measured to determine GSH-t levels (GSH + GSSG). The final product was analyzed in a spectrophotometer at 412 nm. In this process, GSSG was recycled into GSH by GR activity.^32 , 33^

TEAC levels were determined using plasma spectrophotometry and an adapted protocol.^22^ The TEAC assay depends on plasma antioxidant capacity to inhibit 2,2′-azino-bis(3-ethylbenzthiazoline-6-sulfonic acid) oxidation as measured by absorbance at 734 nm. An adapted protocol was used for the plasma MDA analysis.^18^ The high-performance liquid chromatography reading was performed at 533 nm.

This study was approved by the local ethics committee (ethics evaluation submission [CAAE] registry number 20529913.7.0000.5415). All of the subjects were informed about the study and agreed to participate by signing an informed consent form.

### Statistical analysis

Qualitative variables were analyzed using Fisher’s exact test, while quantitative variables were analyzed using ANOVA and Student’s t-test. The Kruskal-Wallis test and the Mann-Whitney test were applied to the non-parametric variables involved in oxidative stress. The significance level was set at 0.05.

## Results

The patient groups (G1 and G2) were similar in terms of age, gender, and PANSS scores (p > 0.05; Table 1 ). Relative to the controls, the patient groups exhibited higher rates of smoking habit (G1 = 27%; G2 = 30%; G1 + G2 = 28%; G3 = 3%; p < 0.05) and of family history of schizophrenia (G1 = 65%; G2 = 56%; G1 + G2 = 62%; G3 = 3%; p < 0.05). However, the groups did not vary in terms of rates of alcohol consumption (p > 0.05).

**Table 1:** Clinical and sociodemographic profile of treatment-responsive schizophrenia patients (G1), treatment-resistant schizophrenia patients (G2), and healthy controls (G3)

	G1 (N = 26)	G2 (N = 27)	G3 (N = 36)	p-value
Age (years)	43 (13.1)	45 (11.1)	39 (13.8)	0.1469* **•**
Age at onset of the disease (years)	25 (11.9)	22 (10.6)	-	0.3010^†^
PANSS				
Positive	13.4 (8.4)	10.5 (4.1)	-	0.5293^†^
Negative	19.5 (10.3)	20.6 (8.7)	-	0.6848^†^
General psychopathology	31.0 (12.4)	27.6 (12.1)	-	0.3688^†^

Data presented as mean (standard deviation).

PANSS = Positive and Negative Syndrome Scale.

* ANOVA test; ^†^ *t* test.

**Figure 1:**
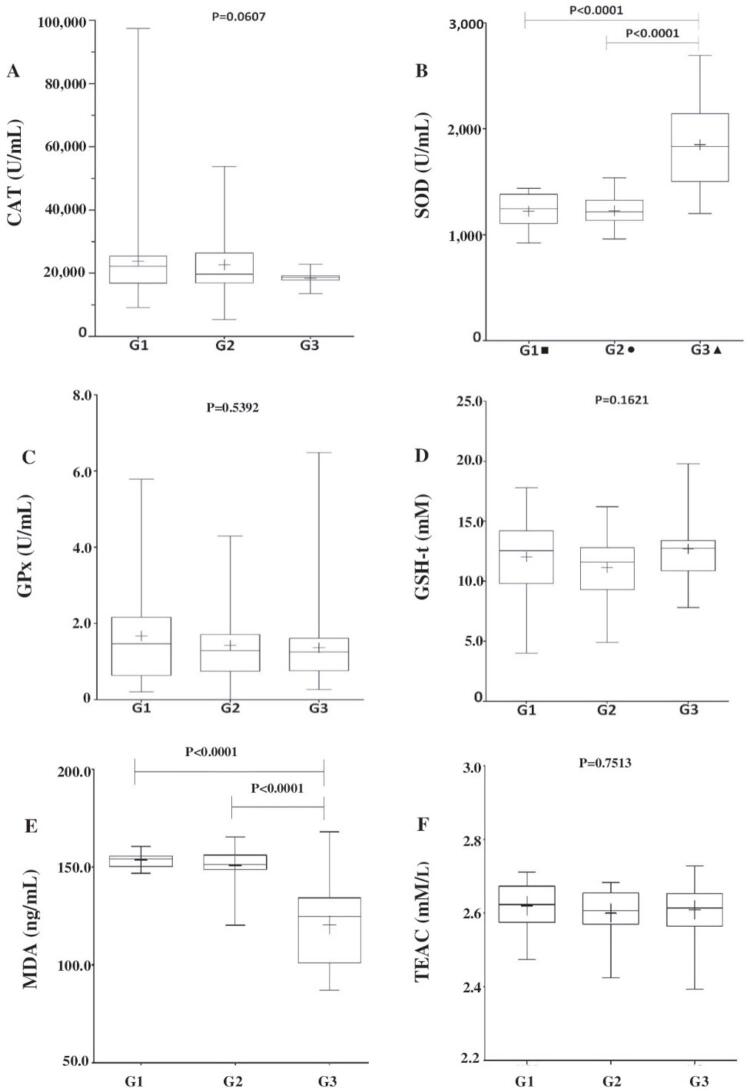
A) Catalase (CAT) values were similar in the patient groups and the control group (p = 0.0607); B) Lower superoxide dismutase (SOD) levels were found in the patient groups (G1 and G2) than in controls (G3; * p < 0.0001); C) Glutathione peroxidase (GPx) levels were similar between groups (p = 0.5392); D) total glutathione (GSH-t) levels were also similar between groups (p = 0.1621). E) Malondialdehyde (MDA) levels were higher in patients (G1 and G2) than in controls (G3; * p < 0.0001). F) Trolox-equivalent antioxidant capacity (TEAC) levels were similar between groups (p = 0.7513). + = mean; – = median; ■: number of treatment-responsive schizophrenia patients = 8; ●: number of treatment-resistant schizophrenia patients = 12; ▲: number of controls = 20; p = significance level according to the Kruskal-Wallis test or* to the Mann-Whitney test.

**Figure 2:**
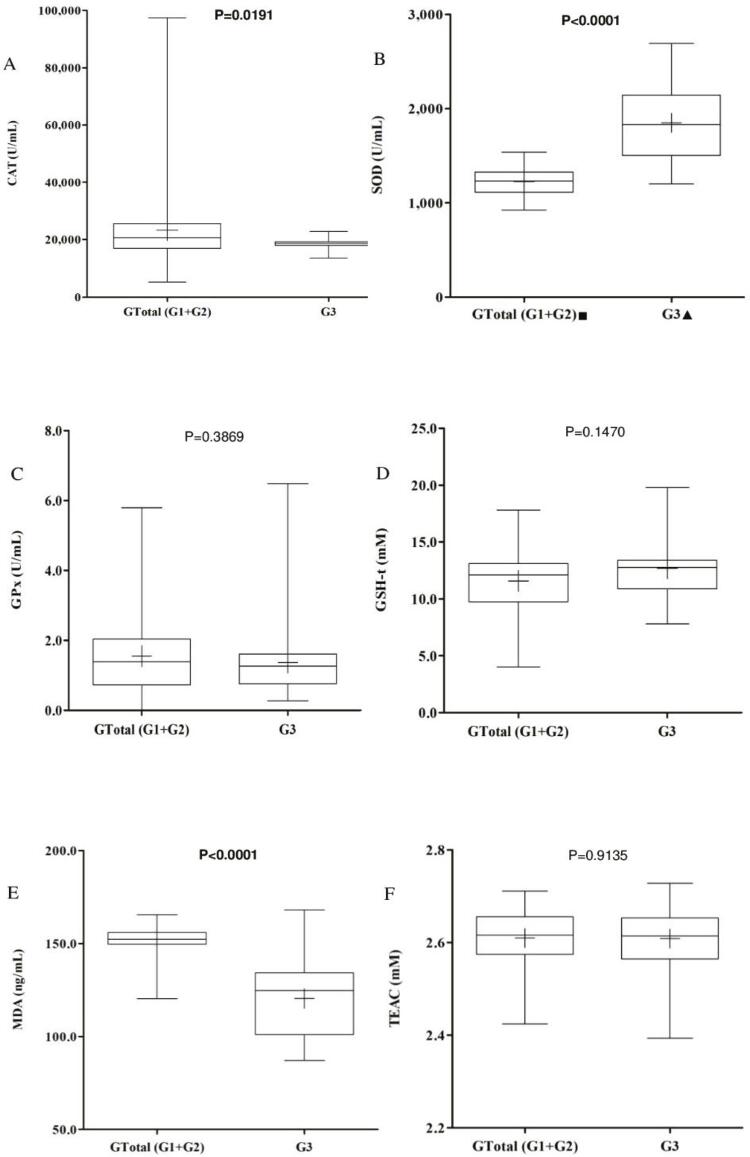
A) Catalase (CAT) values were lower in the control group (G3) than in the two patient groups taken together (G1 + G2; p = 0.0191); B) Lower superoxide dismutase (SOD) levels were found in the patient groups (G1 and G2) than in the controls (G3; * p < 0.0001); C) Glutathione peroxidase (GPx) levels were similar between groups (p = 0.3869); D) Total glutathione (GSH-t) levels were also similar between groups (p = 0.1470). E) Malondialdehyde (MDA) levels were higher in patients (G1 and G2) than in controls (G3; p < 0.0001). F) Trolox-equivalent antioxidant capacity (TEAC) levels were similar between groups (p = 0.9135). + = mean; – = median; ■: number of treatment-responsive schizophrenia patients = 8; ●: number of treatment-resistant schizophrenia patients = 12; ▲: number of controls = 20; p = significance level according to the Mann-Whitney test.

CAT, GPx, GSH-t, and TEAC levels were between the groups (G1 vs. G3; G2 vs. G3; p > 0.05). When both groups of patients together were compared to the controls, the median CAT level for the patient groups was found to be significantly higher than that of the control group (median G1 + G2 = 20,592 U/mL; G3 = 18,662 U/mL; p = 0.0191). However, median SOD values were significantly lower in patients than in controls (G1 = 1,245.0 U/mL; G2 = 1,215.4 U/mL; G1 + G2 = 1,230.8 U/mL; G3 = 1,831.1 U/mL; p < 0.0001), and median MDA levels were significantly higher (G1 = 154.2 ng/mL; G2 = 151.4 ng/mL; G1 + G2 = 152.31ng/mL; G3 = 124.8 ng/mL; p < 0.0001). Details are provided in Figures 1 and 2 .

In the logistic regression analysis (logit A = -0.900189 + 2.693489 smoking + 1.008261 alcohol consumption + 4.081324 family history + 0.017918 gender), significant values were obtained for the independent variables smoking habit (p = 0.0187) and family history of the disease (p = 0.0001), being predictors of development of schizophrenia. Neither alcohol consumption nor gender were found to be predictors of development of schizophrenia (0.9640 and 0.9775, respectively).

## Discussion

This study evaluated oxidative stress markers in treatment-resistant and treatment-responsive schizophrenia patients and found the disease to be associated with lower SOD levels and higher MDA levels, regardless of antipsychotic treatment. These results are consistent with those of other studies performed on both treated and chronic schizophrenia patients,^18^ as well as in studies performed on treatment-naive schizophrenia patients.^34^ It is important to emphasize the efficacy of follow-up and of antipsychotic treatment that rendered the patient groups homogenous. A prior study has reported that these drugs contribute to improved cognitive behavior.^35^ It is also important to note the similar PANSS symptomology between the treatment-resistant and treatment-responsive patients studied herein.

The patients also exhibited a greater rate of smoking relative to the controls, as has been reported in other studies.^36^ Some cigarette ingredients generate the superoxide radical, which may inactivate or reduce SOD levels.^37^ A similar condition may have been present in the patients studied herein and may have contributed to the reduction in SOD levels^38 - 40^ and the consequently higher oxidative damage. This hypothesis may be supported by the high levels of lipid peroxidation represented by MDA levels in schizophrenia patients.^16 , 18^

Antioxidant levels may vary depending on the severity and on the oxidative process of schizophrenia itself.^38 , 41^ Thus, when CAT levels were compared between all of the schizophrenia patients treated as a single group (G1 + G2) and the healthy controls (G3), the enzyme was found to be higher among the patients. There are conflicting reports on CAT activity in schizophrenia patients and these reports include reduced CAT levels,^42^ increased CAT levels, and unchanged CAT levels.^38^ We obtained no significant differences in GPx or GSH-t between patients and controls. Similarly, other studies have reported consistent GPx levels between schizophrenia patients and controls.^42^ However, lower GPx levels,^43 , 44^ GSH-t levels,^42^ and TEAC levels (particularly in patients using atypical antipsychotics)^22^ have been reported by other researchers.

It is important to note the major role that CAT, SOD, GSH, and GPx play in protecting the brain against ROS toxicity.^34 , 45^ However, many factors contribute to the inconsistency of the results presented in the literature, including analysis techniques, the biological samples used, exposure to antipsychotic treatment, and stage of disease, all of which make it difficult to make comparisons between studies. Furthermore, ethnicity, lifestyle, and diet are all likely to produce discrepencies,^38^ as are genetic factors.^46^

This study found a substantial prevalence of a family history of schizophrenia in both patient groups. Another study reported a family history of mental illness in 38.4% and 46.1% of treatment-resistant patients and treatment-responsive patients, respectively,^47^ a finding which gives weight to the influence of genetics on the development of schizophrenia reported in the literature.^8 , 48^ Although the disease has a strong hereditary basis, its multifactorial etiology leaves much to be investigated. The small sample size used herein was a result of the specific inclusion criteria and of the need for a homogenous patient profile and may be cited as a limitation of this study. However, this study included more subjects than others,^18 , 22^ and there were enough participants to allow for statistical analysis of the data.

Analyses were not performed to determine any correlations between the oxidative stress biomarkers and the antipsychotics used by the patients included herein. It is known that typical and atypical antipsychotics may influence oxidative stress in different ways.^43 , 49 , 50^ Information on the criteria used to define patients as treatment-resistant were obtained during the early stages of the study; this information included medical records, as well as patients’, family members’, and caregivers’ reports on patients’ use of and compliance with antipsychotics. Stricter criteria, including the use of long-acting injectable antipsychotics and tests of antipsychotic serum levels, have been suggested as ways to more accurately establish cases of treatment-resistant schizophrenia.^51^ Methods such as these help prevent issues such as failed compliance with treatment, poor medication absorption, and pharmacological interactions, any of which may produce inadequate treatment or prevent correct classification of these patients.

In conclusion, increased MDA and CAT levels and decreased SOD levels indicate that schizophrenia patients are exposed to oxidative stress. Furthermore, the prevalence of a family history of the disease in the patient groups supports the involvement of genetic factors in the development of the disease, regardless of treatment resistance. Therefore, the role of the oxidative pathway as either a cause or consequence of schizophrenia still requires clarification. This cross-sectional study’s findings on the association between oxidative stress biomarkers and schizophrenia may contribute to future longitudinal studies that seek to prove causal relationships between neurodegenerative processes, the severity of psychotic symptoms, and resistance to treatment.

## References

[1] 1. World Health Organization (WHO). Mental disorders. 2018. [cited Apr 2019]. www.who.int/mediacentre/factsheets/fs396/en/

[2] 2. Etemadikhah M, Niazi A, Wetterberg L, Feuk L. Transcriptome analysis of fibroblasts from schizophrenia patients reveals differential expression of schizophrenia-related genes. Sci Rep. 2020;10:630. 10.1038/s41598-020-57467-z PMC6971273 31959813

[3] 3. Németh B, Fasseeh A, Molnár A, Bitter I, Horváth M, Kóczián K, et al. A systematic review of health economic models and utility estimation methods in schizophrenia. Expert Rev Pharmacoecon Outcomes Res. 2018;18:267-75. 10.1080/14737167.2018.1430571 29347854

[4] 4. Carrà G, Crocamo C, Bartoli F, Angermeyer M, Brugha T, Toumi M, et al. The mediating role of depression in pathways linking positive and negative symptoms in schizophrenia. A longitudinal analysis using latent variable structural equation modelling. Psychol Med. 2020;50:566-74. 10.1017/S0033291719000321 30846005

[5] 5. Hirayasu Y, Sato S, Shuto N, Nakano M, Higuchi T. Efficacy and safety of bitopertin in patients with schizophrenia and predominant negative symptoms: subgroup analysis of Japanese patients from the global randomized phase 2 trial. Psychiatry Investig. 2017;14:63-73. 10.4306/pi.2017.14.1.63 PMC5240458 28096877

[6] 6. Kaliuzhna M, Kirschner M, Carruzzo F, Hartmann-Riemer MN, Bischof M, Seifritz E, et al. Clinical, behavioural and neural validation of the PANSS amotivation factor. Schizophr Res. 2020;220:38-45. 10.1016/j.schres.2020.04.018 32349887

[7] 7. Saliba M, Assaad S, Haddad C, Hallit S, Hachem D, Haddad G. Schizophrenia and smoking: impact on negative symptoms. Rev Int Investig Adicciones. 2017;3.28-35.

[8] 8. Käkelä J, Marttila R, Keskinen E, Veijola J, Isohanni M, Koivumaa-Honkanen H, et al. Association between family history of psychiatric disorders and long-term outcome in schizophrenia - the Northern Finland birth cohort 1966 study. Psychiatry Res. 2017;249:16-22. 10.1016/j.psychres.2016.12.040 28063393

[9] 9. Amiaz R, Rubinstein K, Czerniak E, Karni Y, Weiser M. A Diet and fitness program similarly affects weight reduction in schizophrenia patients treated with typical or atypical medications. Pharmacopsychiatry. 2016;49:112-6. 10.1055/s-0035-1569416 26909490

[10] 10. Krause D, Pogarell O. Shrinking brain and schizophrenia: a review of current studies on the effect of antipsychotic medication on gray matter volume. Psych Mental Disord. 2017;1:102.

[11] 11. Gillespie AL, Samanaite R, Mill J, Egerton A, MacCabe JH. Is treatment-resistant schizophrenia categorically distinct from treatment-responsive schizophrenia? A systematic review. BMC Psychiatry. 2017;17:12. 10.1186/s12888-016-1177-y PMC5237235 28086761

[12] 12. Polho GB, Paula VJ. Schizophrenia: neuroinflammation, neurodegeneration or neurodevelopment? A genetic overview. Rev Med 2017;96:39-48.

[13] 13. Xie ZZ, Liu Y, Bian JS. Hydrogen sulfide and cellular redox homeostasis. Oxid Med Cell Longev. 2016;2016:6043038. 10.1155/2016/6043038 PMC4736422 26881033

[14] 14. Kruk J, Aboul-Enein HY, Kładna A, Bowser JE. Oxidative stress in biological systems and its relation with pathophysiological functions: the effect of physical activity on cellular redox homeostasis. Free Radic Res. 2019;53:497-521. 10.1080/10715762.2019.1612059 31039624

[15] 15. Sghaier R, Zarrouk A, Nury T, Ilham B, O’Brien N, Mackrill JJ, et al. Biotin attenuation of oxidative stress, mitochondrial dysfunction, lipid metabolism alteration and 7β-hydroxycholesterol-induced cell death in 158N murine oligodendrocytes. Free Radic Res. 2019;53:535-61. 10.1080/10715762.2019.1612891 31039616

[16] 16. Zheng W, Zhu W, Feng Z, Liang Q, Rong PF, Li LF, et al. Increased serum malondialdehyde levels are associated with grey matter volume loss in patients with non-alcoholic cirrhosis. Quant. Imaging Med Surg. 2019;9:230-7. 10.21037/qims.2018.12.12 PMC6414766 30976547

[17] 17. Filho GM, Martins DP, Lopes AM, Brait BJ, Furlan AE, Oliveira CI, et al. Oxidative stress in patients with refractory temporal lobe epilepsy and mesial temporal sclerosis: possible association with major depressive disorder. Epilepsy Behav. 2018;80:191-6. 10.1016/j.yebeh.2017.12.025 29414551

[18] 18. Liencres CG, Tas C, Brown EC, Erdin S, Onur C, Cubukcoglu Z, et al. Oxidative stress in schizophrenia: a case–control study on the effects on social cognition and neurocognition. BMC Psychiatry. 2014;14:268. 10.1186/s12888-014-0268-x PMC4180831 25248376

[19] 19. Liu W, Yang T, Xu Z, Xu B, Deng Y. Methyl-mercury induces apoptosis through ROS-mediated endoplasmic reticulum stress and mitochondrial apoptosis pathways activation in rat cortical neurons. Free Radic Res. 2019;53:26-44. 10.1080/10715762.2018.1546852 30513015

[20] 20. Pawar MRS and Abhang AS. Consumption of GSH with the increase in oxidative stress in chronic obstructive pulmonary disease (COPD) patients. Int J Cur Res Rev. 2017;9:25-9.

[21] 21. Forner L, Andreguetto TN, Lopes AM, Buosi P, Borghi FA. Facincani IS, et al. Genetic and biochemical biomarkers related to oxidative stress in patients with schizophrenia. Genet Mol Res. 2019;18:GMR18259.

[22] 22. Gilca M, Piriu G, Gaman L, Delia C, Iosif L, Atanasiu V, et al. A study of antioxidant activity in patients with schizophrenia taking atypical antipsychotics. Psychopharmacology. 2014;231:4703-10. 10.1007/s00213-014-3624-0 PMC4231214 24871701

[23] 23. Campos KKD, Araújo GR, Martins TL, Bandeira ACB, Costa GP, Talvani A, et al. The antioxidant and anti-inflammatory properties of lycopene in mice lungs exposed to cigarette smoke. J Nutr Biochem. 2017;48:9-20. 10.1016/j.jnutbio.2017.06.004 28651168

[24] 24. Bhagat J, Ingole BS, Singh N. Glutathione S-transferase, catalase, superoxide dismutase, glutathione peroxidase, and lipid peroxidation as biomarkers of oxidative stress in snails: a review. 2016;13:336-49.

[25] 25. American Psychiatric Association. Diagnostic and Statistical Manual of Mental Disorders, Fourth Edition, Text Revision (DSM-IV-TR). Arlington: American Psychiatric Publishing; 2000.

[26] 26. Kane J, Honigfeld G, Singer J, Meltzer H. Clozapine for the treatment-resistant schizophrenic. A double-blind comparison with chlorpromazine. Arch Gen Psychiatry. 1988;45:789-96. 10.1001/archpsyc.1988.01800330013001 3046553

[27] 27. Worl Health Organization (WHO). The international psychopharmacology algorithm project. [Internet]: 2015 [cited 2021 Jul 13]. www.ipap.org

[28] 28. Kay SR, Fiszbeln A, Opfer, LA. The positive and negative syndrome scale (PANSS) for schizophrenia. Schizophr Bull. 1967;13:261-76. 10.1093/schbul/13.2.261 3616518

[29] 29. Mccord JM, Fridovich I. Superoxide dismutase. An enzymic function for erythrocuprein (hemocuprein). J Biol Chem. 1969;244:6049-55. 5389100

[30] 30. Beutler E. Red cell metabolism: a manual of biochemical methods. New York: Grune & Stratton; 1975.

[31] 31. Sies H, Koch OR, Martino E, Boveris A. Increased biliary glutathione disulfide release in chronically ethanol-treated rets. Febs Lett. 1979;103:287-90. 10.1016/0014-5793(79)81346-0 467672

[32] 32. Tietze F. Enzymic method for quantitative determination of nanogram amounts of total and oxidized glutathione: applications to mammalian blood and other tissues. Anal Biochem. 1969;27:502-22. 10.1016/0003-2697(69)90064-5 4388022

[33] 33. Akerboom TP, Sies H. Assay of glutathione disulfide and glutathione mixed disulfide in biological samples. Meth. Enzymol. 1981;77:373-82. 10.1016/s0076-6879(81)77050-2 7329314

[34] 34. Reyazuddin M, Azmi, SA, Islam N, Rizvi A. Oxidative stress and level of antioxidant enzymes in drug-naive schizophrenics. Indian J Psychiatry. 2014;56:344-9. 10.4103/0019-5545.146516 PMC4279291 25568474

[35] 35. Rajagopal L, Kwon S, Huang M, Michael E, Bhat L, Cantillon M et al. RP5063, an atypical antipsychotic drug with a unique pharmacologic proﬁle, improves declarative memory and psychosis in mouse models of schizophrenia. Behav Brain Res. 2017;332:180-99. 10.1016/j.bbr.2017.02.036 28373127

[36] 36. Jordan W, Dobrowolny H, Bahn S, Bernstein HG, Brigadski T, Frod T, et al. Oxidative stress in drug-naïve first episode patients with schizophrenia and major depression: effects of disease acuity and potential confounders. Eur Arch Psychiatry Clin Neurosci. 2018;268:129-43. 10.1007/s00406-016-0749-7 27913877

[37] 37. Coughlin JM, Hayes LN, Tanaka T, Xiao M, Yolken RH, Worley P, et al. Reduced superoxide dismutase-1 (SOD1) in cerebrospinal fluid of patients with early psychosis in association with clinical features. Schizophr Res. 2017;183:64-9. 10.1016/j.schres.2016.10.040 27889384

[38] 38. Zhang XY, Tan YL, Cao LY, Wu GY, Xu Q, Shen Y, Zhou DF. Antioxidant enzymes and lipid peroxidation in different forms of schizophrenia treated with typical and atypical antipsychotics. Schizophr Res. 2006;81:291-300. 10.1016/j.schres.2005.10.011 16309894

[39] 39. Pasupathi P, Saravanan G, Farook J. Oxidative Stress Bio Markers and Antioxidant Status in Cigarette Smokers Compared to Nonsmokers. J Pharm Sci Res. 2009;1:55-62.

[40] 40. Avval FZ, Mahmoudi N, Tirkani AN, Jarahi L, Alamdari DH, Sadjadi SA. Determining pro-oxidant antioxidant balance (PAB) and total antioxidant capacity (TAC) in patients with schizophrenia. Iran J Psychiatry. 2018;13:222-6. PMC6178326 30319706

[41] 41. Padurariu M, Ciobica A, Dobrin I, Stefanescu C. Evaluation of antioxidant enzymes activities and lipid peroxidation in schizophrenic patients treated with typical and atypical antipsychotics. Neurosci Lett. 2010;479:317-20. 10.1016/j.neulet.2010.05.088 20561936

[42] 42. Raffa M, Mechri A, Othman LB, Fendri C, Gaha L, Kerkeni A. Decreased glutathione levels and antioxidant enzyme activities in untreated and treated schizophrenic patients. Prog Neuropsychopharmacol Biol Psychiatry. 2009;33:1178-83. 10.1016/j.pnpbp.2009.06.018 19576938

[43] 43. Dietrich-Muszalska A, Kwiatkowska A. Generation of superoxide anion radicals and platelet glutathione peroxidase activity in patients with schizophrenia. Neuropsychiatr Dis Treat. 2014;10:703-9. 10.2147/NDT.S60034 PMC4015795 24833903

[44] 44. Yao JK, Leonard S, Reddy R. Altered glutathione redox state in schizophrenia. Dis Markers. 2006; 22:83-93. 10.1155/2006/248387 PMC3850558 16410648

[45] 45. Bai X, Fermandez E, Gould G, Strong R. Homozygous. Deletion of glutathione peroxidase 1 and aldehyde dehydrogenase 1a1 genes is not associated with schizophrenia-like behavior in mice. J Biochem Pharmacol Res. 2013;1:228-35. PMC3909525 24494173

[46] 46. Ballesteros A, Jiang P, Summerfelt A, Du X, Chiappelli J, O’Donnell P. No evidence of exogenous origin for the abnormal glutathione redox state in schizophrenia. Schizophr Res. 2013;146:184-9. 10.1016/j.schres.2013.02.001 PMC3622807 23466187

[47] 47. Medina-Hernández V, Ramos-Loyo J, Luquin S, Sánchez LF, García-Estrada J, Navarro-Ruiz A. Increased lipid peroxidation and neuron specific enolase in treatment refractory schizophrenics. J Psychiatr Res. 2007;41:652-8. 10.1016/j.jpsychires.2006.02.010 16600300

[48] 48. Giusti-Rodríguez P, Sullivan PF. The genomics of schizophrenia: update and implications. J Clin Invest. 2013;123:4557-63. 10.1172/JCI66031 PMC3809776 24177465

[49] 49. Güneş M, Camkurt MA, Bulut M, Demir S, İbiloğlu AO, Kaya MC, et al. Evaluation of paraoxonase, arylesterase and malondialdehyde levels in schizophrenia patients taking typical, atypical and combined antipsychotic treatment. Clin Psychopharmacol Neurosci. 2016;14:345-50. 10.9758/cpn.2016.14.4.345 PMC5083945 27776386

[50] 50. Ficarra S, Russo A, Barreca D, Giunta E, Galtieri A, Tellone E. Short-term effects of chlorpromazine on oxidative stress in erythrocyte functionality: activation of metabolism and membrane perturbation. Oxid Med Cell Longev. 2016;2016:2394130. 10.1155/2016/2394130 PMC4992801 27579150

[51] 51. Howes OD, McCutcheon R, Agid O, de Bartolomeis A, van Beveren NJ, Birnbaum ML, et al. Treatment-resistant schizophrenia: treatment response and resistance in psychosis (TRRIP) working group consensus guidelines on diagnosis and terminology. Am J Psychiatry. 2017;174:216-29. 10.1176/appi.ajp.2016.16050503 PMC6231547 27919182

